# Supplementary material for: Prevalence of Electrocardiographic Abnormalities in Patients with Acute Pulmonary Embolism: A Systematic Review and Meta-Analysis
Source: J Clin Med. 2025 Jul 4;14(13):4750. doi: 10.3390/jcm14134750 (PMC12251390; doi:10.3390/jcm14134750)

Supplementary Appendix

Prevalence of Electrocardiographic Abnormalities in Patients with Acute Pulmonary Embolism: A Systematic Review and Meta-analysis

Journal of Clinical Medicine

Table S1. Details of electronic search terms

Database		Search terms	Results
**PubMed**	#1	(Prevalence OR Epidemiology OR incidence)	4318637
	#2	"pulmonary embolism"[Mesh]	44290
	#3	"electrocardiography"[Mesh]	218223
	#4	#1 AND #2 AND #3	218
	#5	#4 Filters: Humans	212
**Web of**	#1	pulmonary embolism (All Fields)	57152
**Science**	#2	electrocardiography OR electrocardiogram OR ECG OR EKG	141604
	#3	(All Fields)	13694132
	#4	incidence OR prevalence OR finding OR epidemiology (All	474
		Fields)	
		#1 AND #2 AND #3	
**Embase**	#1	'lung embolism'/exp OR 'chronic lung embolism' OR	141491
		'embolism, lung' OR 'lung embolism' OR 'lung embolization'	
		OR 'lung embolus' OR 'lung embolus recurrence' OR 'lung	
		emboly' OR 'lung microembolism' OR 'lung	
		microembolization' OR 'lung microembolus' OR 'lung	
		thromboembolism' OR 'microembolus, lung' OR 'pulmonary	
		embolism' OR 'pulmonary embolization' OR 'pulmonary	
		embolus' OR 'pulmonary microembolism' OR 'pulmonary	
	#2	thromboembolic disease' OR 'pulmonary thromboembolism'	339189
		OR 'thromboembolism, lung'	
		electrocardiogram/exp OR 'ECG' OR 'cardiogram' OR 'e.c.g.'	
		OR 'ecg analysis' OR 'ekg' OR 'electric cardiogram' OR	
	#3	'electro cardiogram' OR 'electrocardiogram' OR 'isoelectric	1663941
	#4	ecg' OR 'orthogonal ecg' OR 'praecordial ecg' OR 'precordial	522
		ecg'	
		prevalence/exp OR incidence/exp	
		#1 AND #2 AND #3	
**Cochrane**	#1	"pulmonary embolism"[Title Abstract Keyword] Limits to	4461
**Collaboration**	#2	"Trials"	36774
	#3	"electrocardiography"[Title Abstract Keyword] Limits to	93
		"Trials"	
		#1 AND #2	
**Scopus**	#1	"pulmonary embolism" [Title Abstract Keyword]	68181
	#2	"electrocardiogram" OR "electrocardiography" OR "ECG" OR	428825
		"EKG" [Title Abstract Keyword]	
	#3	incidence OR prevalence [Title Abstract Keyword]	2736060
	#4	#1 AND #2 AND #3	414

Table S2. Study's risk of bias using the Joanna Briggs Institute (JBI) critical appraisal checklist for prevalence studies

Study	Was the sample frame appropriate to address the target population?	Were study participants sampled in an appropriate way?	Was the sample size adequate?	Were the study subjects and the setting described in detail?	Was the data analysis conducted with sufficient coverage of the identified sample?	Were valid methods used for the identification of the condition?	Was the condition measured in a standard, reliable way for all participants?	Was there an appropriate statistical analysis?	Was the response rate adequate, and if not, was the low response rate managed properly?
**Wang, 2023**	Yes	Yes	Unclear	Yes	Yes	Yes	Yes	Yes	Yes
**Kusayama, 2019**	Yes	Yes	Unclear	Yes	Yes	Yes	Yes	Yes	Yes
**Pourafkari, 2017**	Yes	Yes	Yes	Yes	Yes	Yes	Yes	Yes	Yes
**Çagdas, 2018**	Yes	Yes	Unclear	Yes	Yes	Yes	Yes	Yes	Yes
**Park, 2017**	Yes	Yes	Unclear	Yes	Yes	Yes	Yes	Yes	Yes
**Rodger, 2000**	Unclear	Yes	Unclear	Unclear	Unclear	Yes	Yes	Yes	Yes
**Bahreini, 2024**	Yes	Yes	Yes	Yes	Yes	Yes	Unclear	Yes	Yes
**Thomson, 2019**	Yes	Yes	Unclear	Yes	Yes	Yes	Yes	Yes	Yes
**Zhan, 2014**	Unclear	Yes	Unclear	Yes	Yes	Yes	Yes	Yes	Yes
**Weekes, 2022**	Yes	Yes	Unclear	Yes	Yes	Yes	Yes	Yes	Yes
**Richman, 2004**	Unclear	Yes	Unclear	Yes	Unclear	Yes	Yes	Yes	Yes
**Kukla, 2011**	Yes	Yes	Unclear	Yes	Yes	Yes	Unclear	Yes	Yes
**Novicic, 2020**	Yes	Yes	Unclear	Yes	Yes	Yes	Unclear	Yes	Yes
**Cetin, 2016**	Yes	Yes	Unclear	Yes	Yes	Yes	Yes	Yes	Yes

**Obradovic, 2016**	Yes	Yes	Unclear	Yes	Yes	Yes	Unclear	Yes	Yes
**Ivan, 2017**	Yes	Yes	Unclear	Yes	Yes	Yes	Unclear	Yes	Yes
**Casazza, 2018**	Yes	Yes	Unclear	Yes	Yes	Yes	Yes	Yes	Yes
**Yan, 2024**	Yes	Yes	Unclear	Yes	Yes	Yes	Yes	Yes	Yes
**Vanni, 2009**	Yes	Yes	Yes	Yes	Yes	Yes	Yes	Yes	Yes
**Bolt, 2019**	Yes	Yes	Unclear	Yes	Yes	Yes	Yes	Yes	Yes
**Geibel, 2005**	Yes	Yes	Unclear	Yes	Yes	Yes	Yes	Yes	Yes
**Stein, 2013**	Yes	Yes	Unclear	Yes	Yes	Yes	Unclear	Yes	Yes
**Witting, 2012**	Unclear	Yes	Yes	Unclear	Unclear	Yes	Yes	Yes	Yes
**Zhang, 2016**	Yes	Yes	Unclear	Yes	Yes	Yes	Unclear	Yes	Yes

Figure S1. Forest plot of the prevalence of S1Q3T3 in patients with acute pulmonary embolism

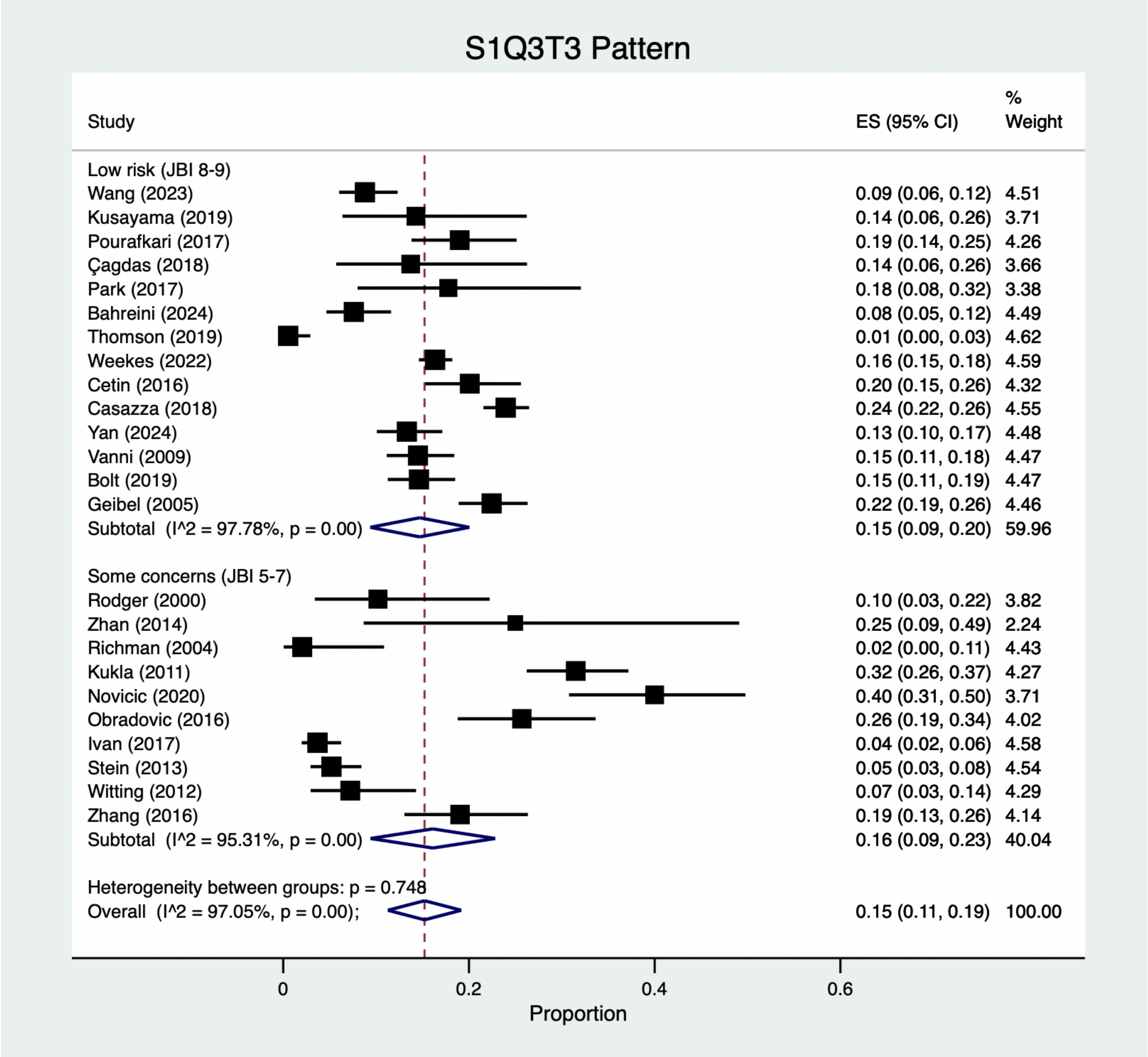

Figure S2. Forest plot of the prevalence of sinus tachycardia in patients with acute pulmonary embolism

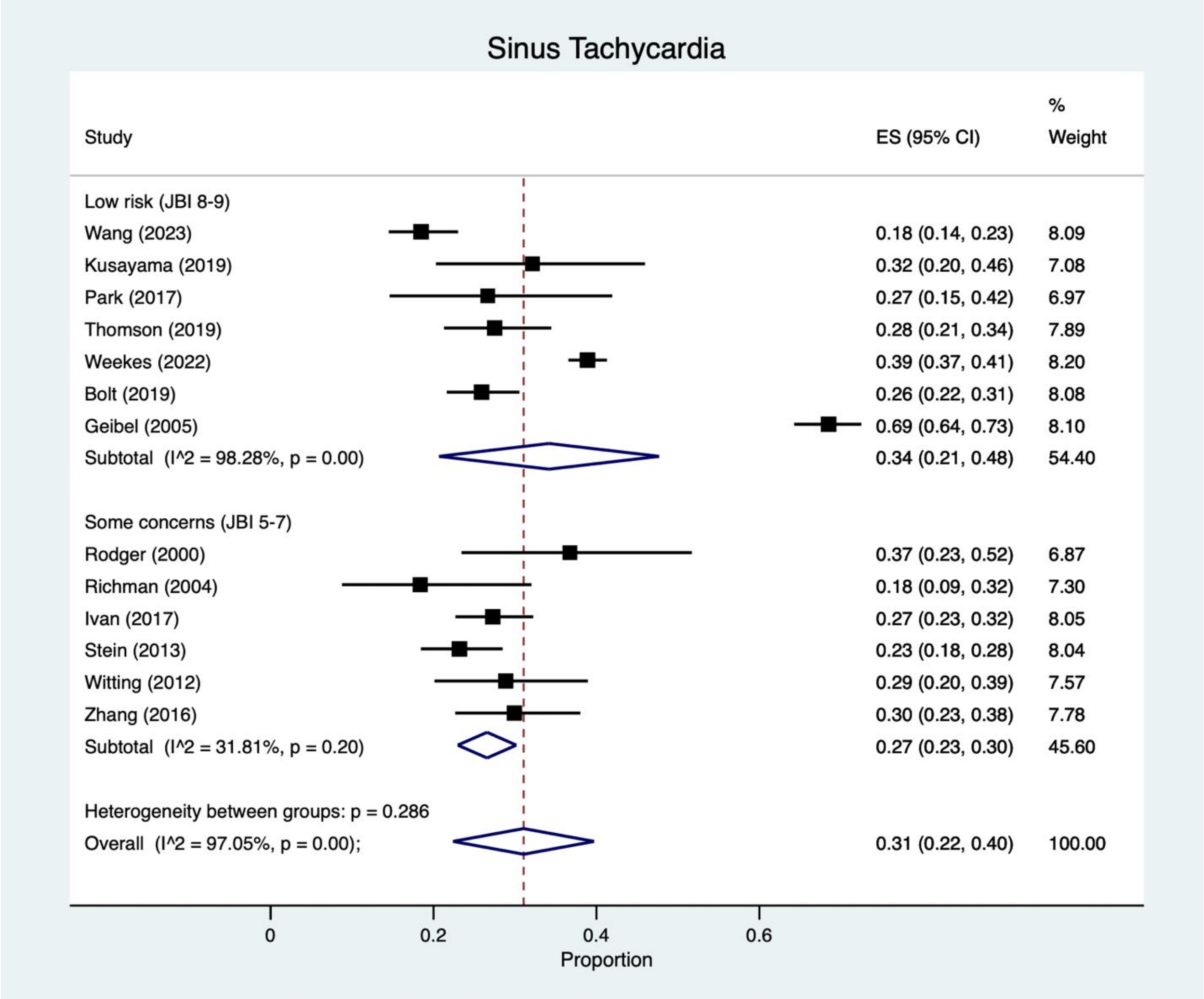

Figure S3. Forest plot of the prevalence of right bundle branch block in patients with acute pulmonary embolism

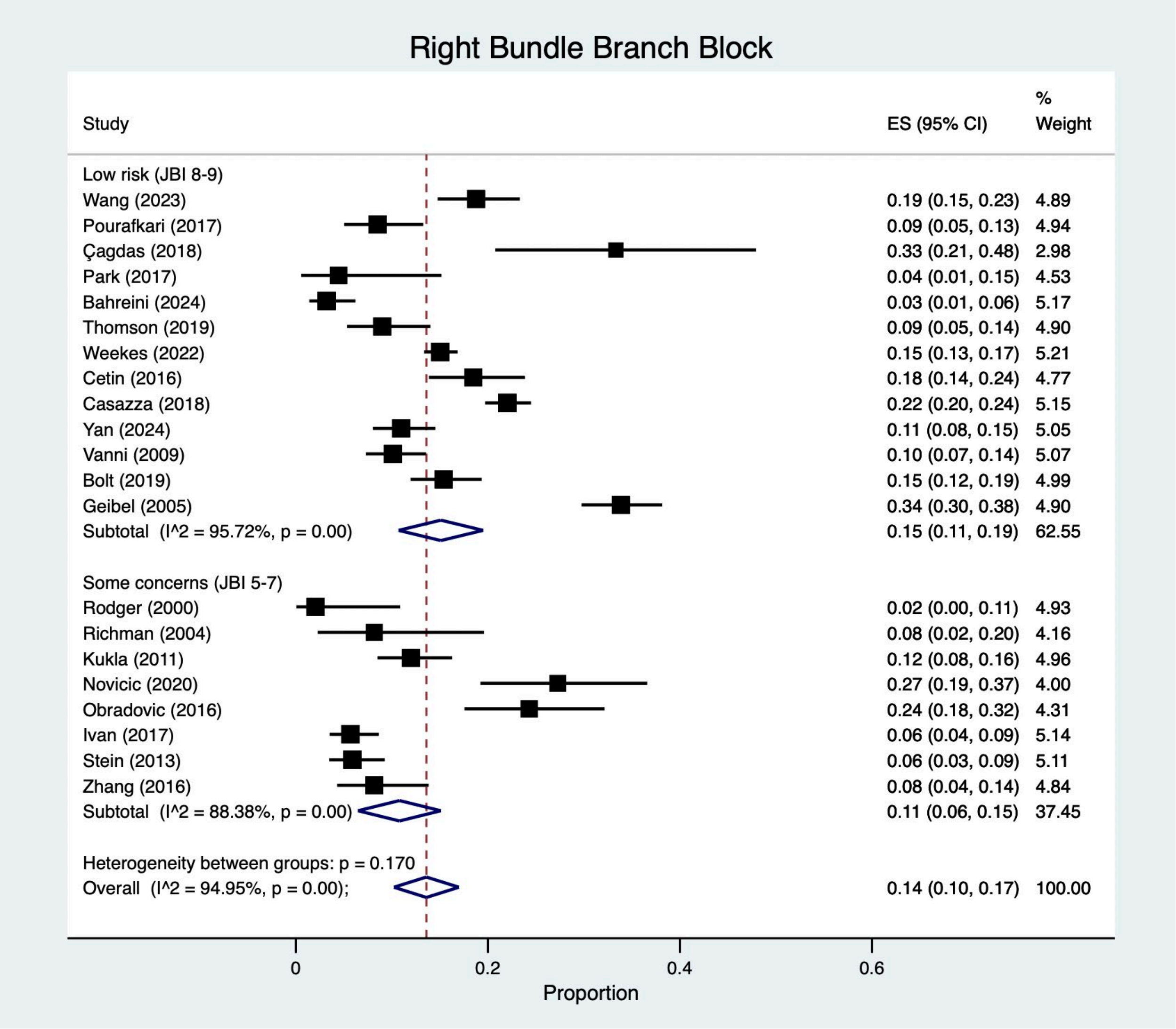

Figure S4. Forest plot of the prevalence of right axis deviation in patients with acute pulmonary embolism

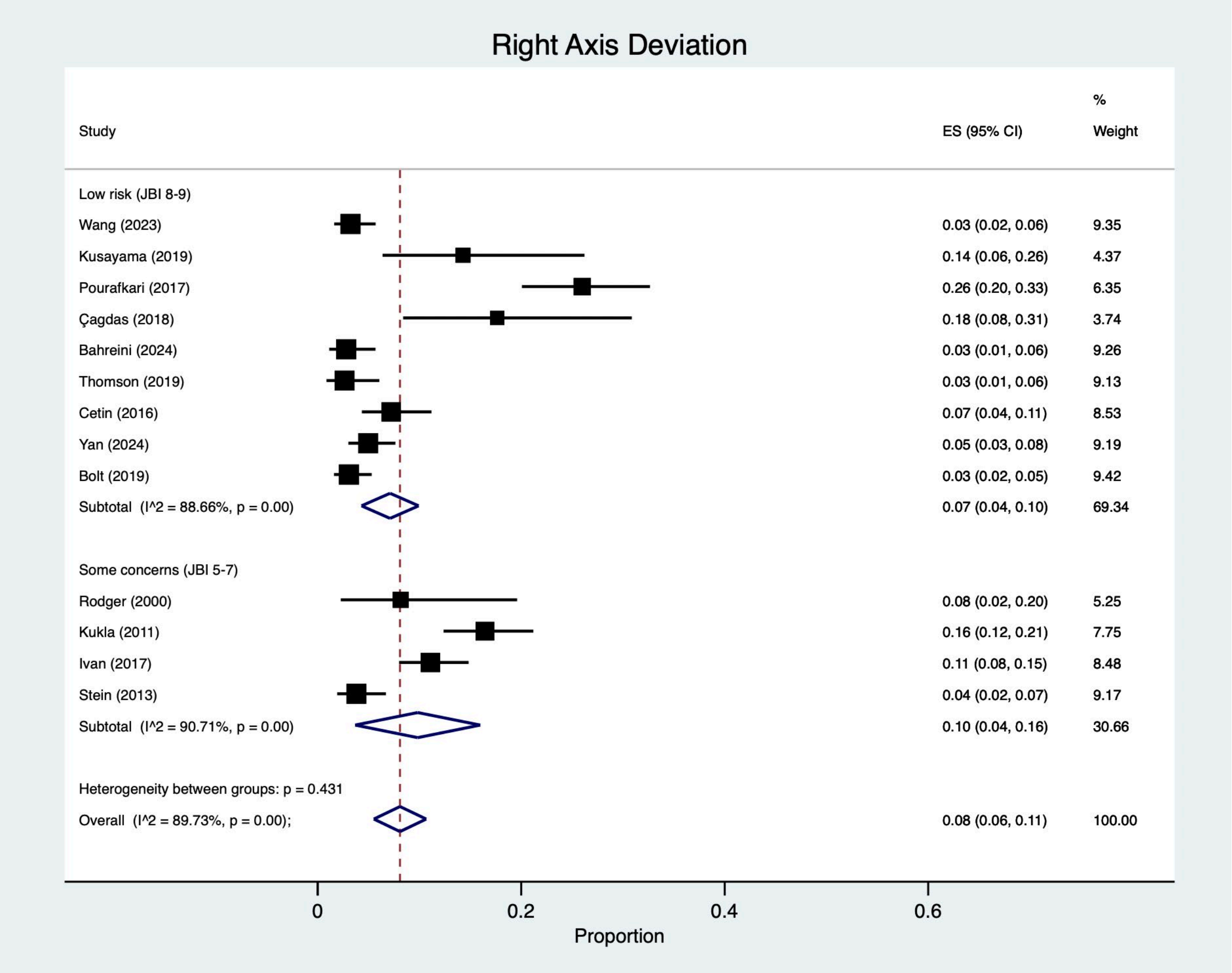

Figure S5. Forest plot of the prevalence of atrial fibrillation/atrial flutter in patients with acute pulmonary embolism

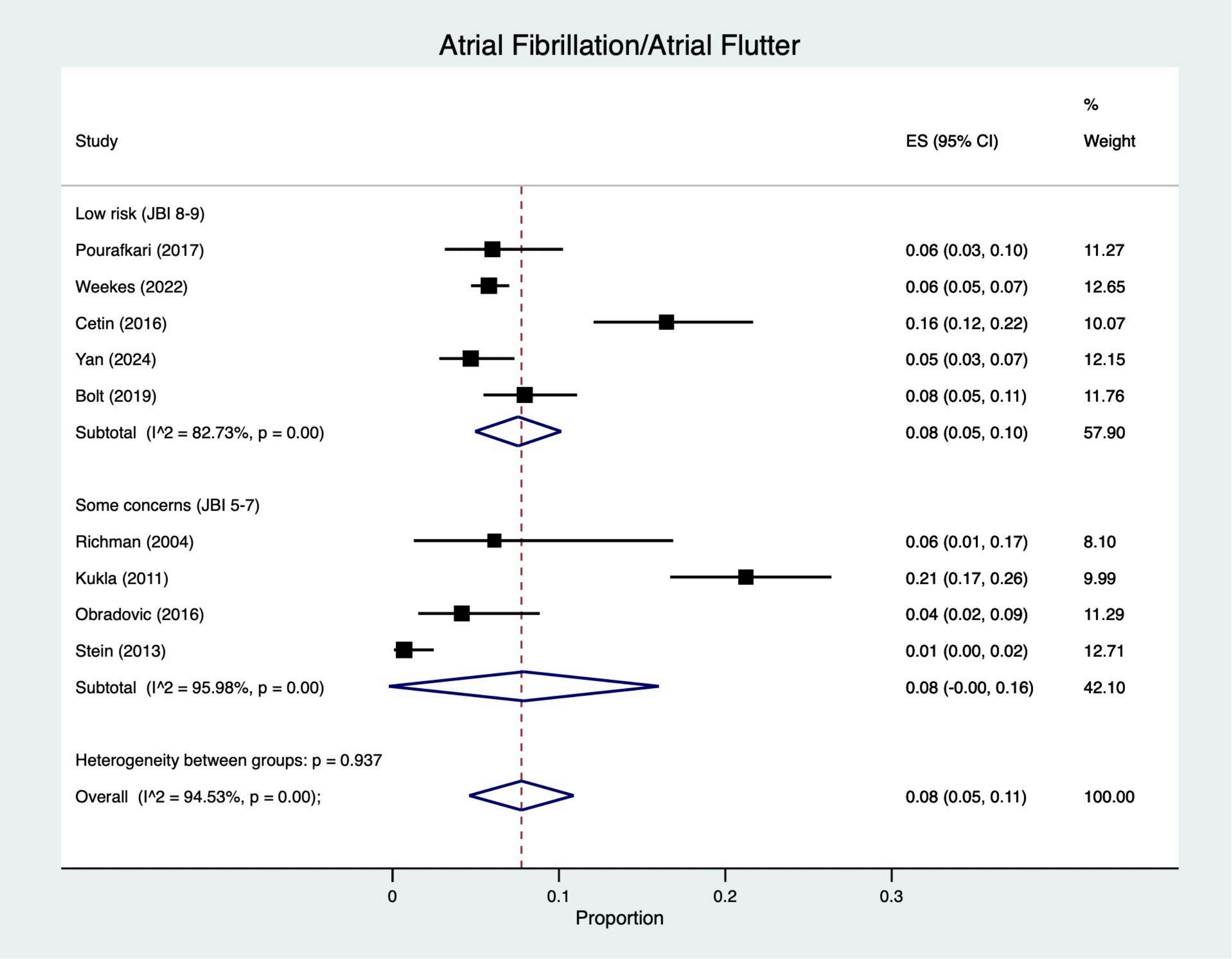

Figure S6. Forest plot of the prevalence of low voltage in patients with acute pulmonary embolism

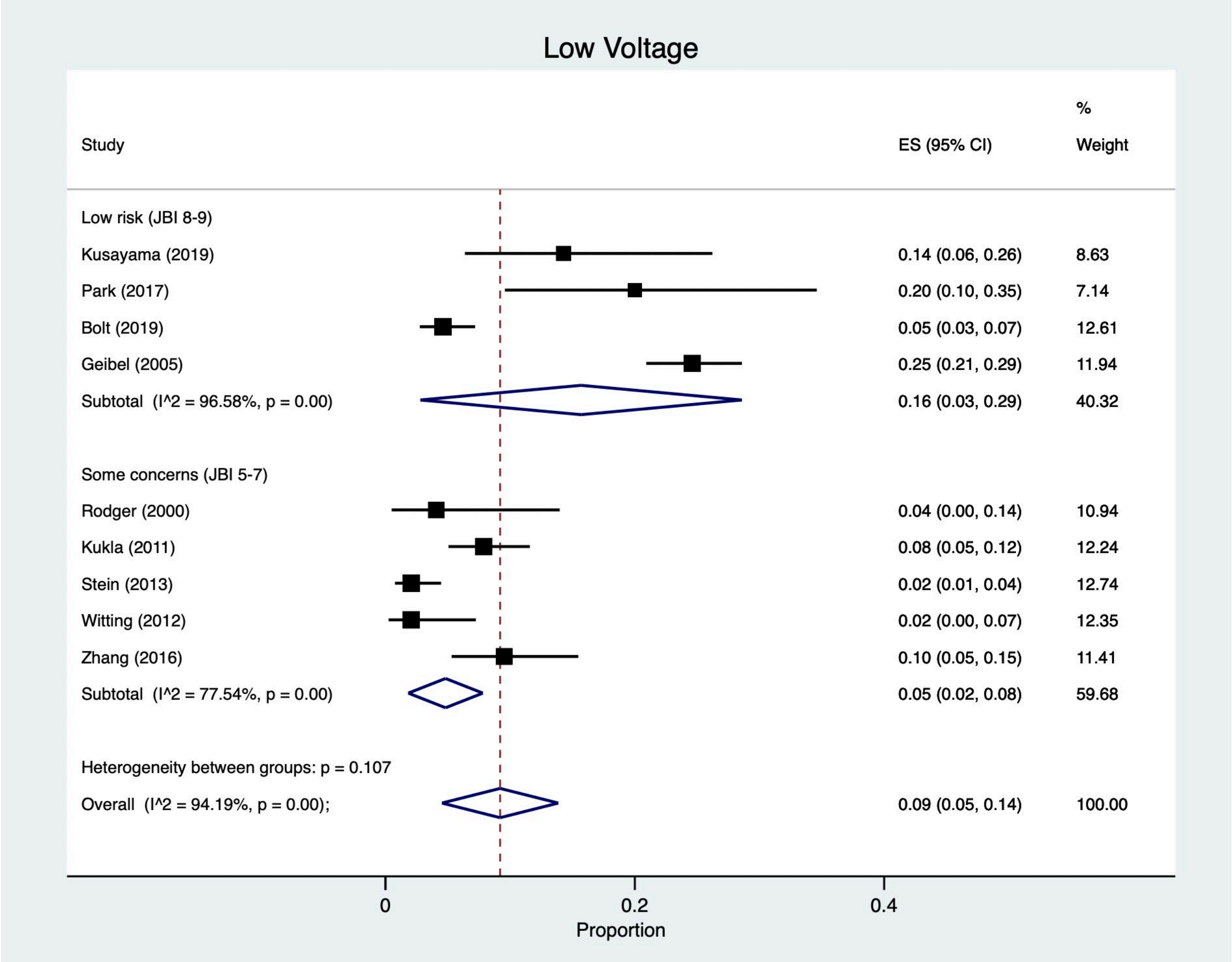

Figure S7. Forest plot of the prevalence of T wave inversion in leads V1-V3 in patients with acute pulmonary embolism

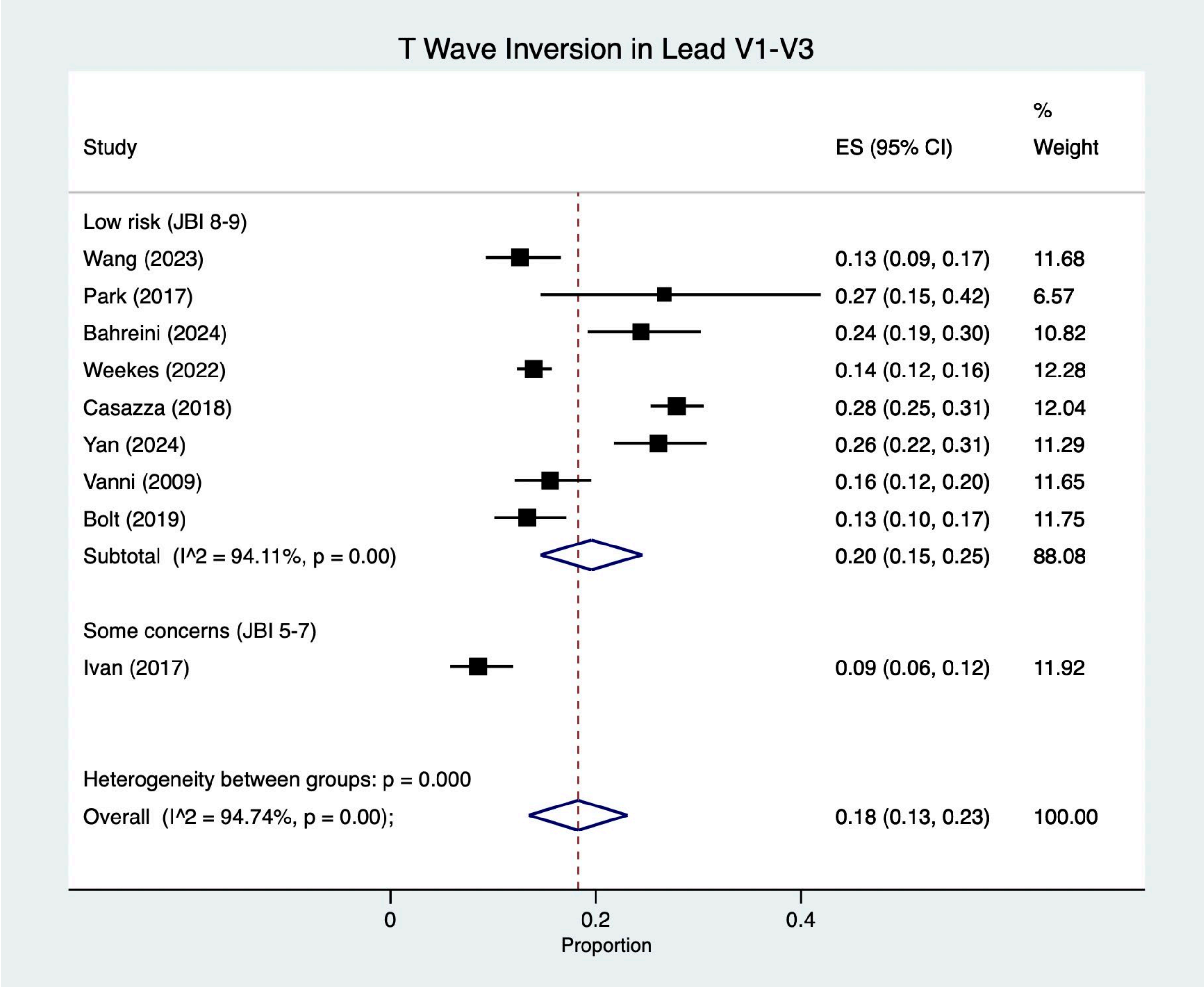

Figure S8. Forest plot of the prevalence of atrial arrhythmia in patients with acute pulmonary embolism

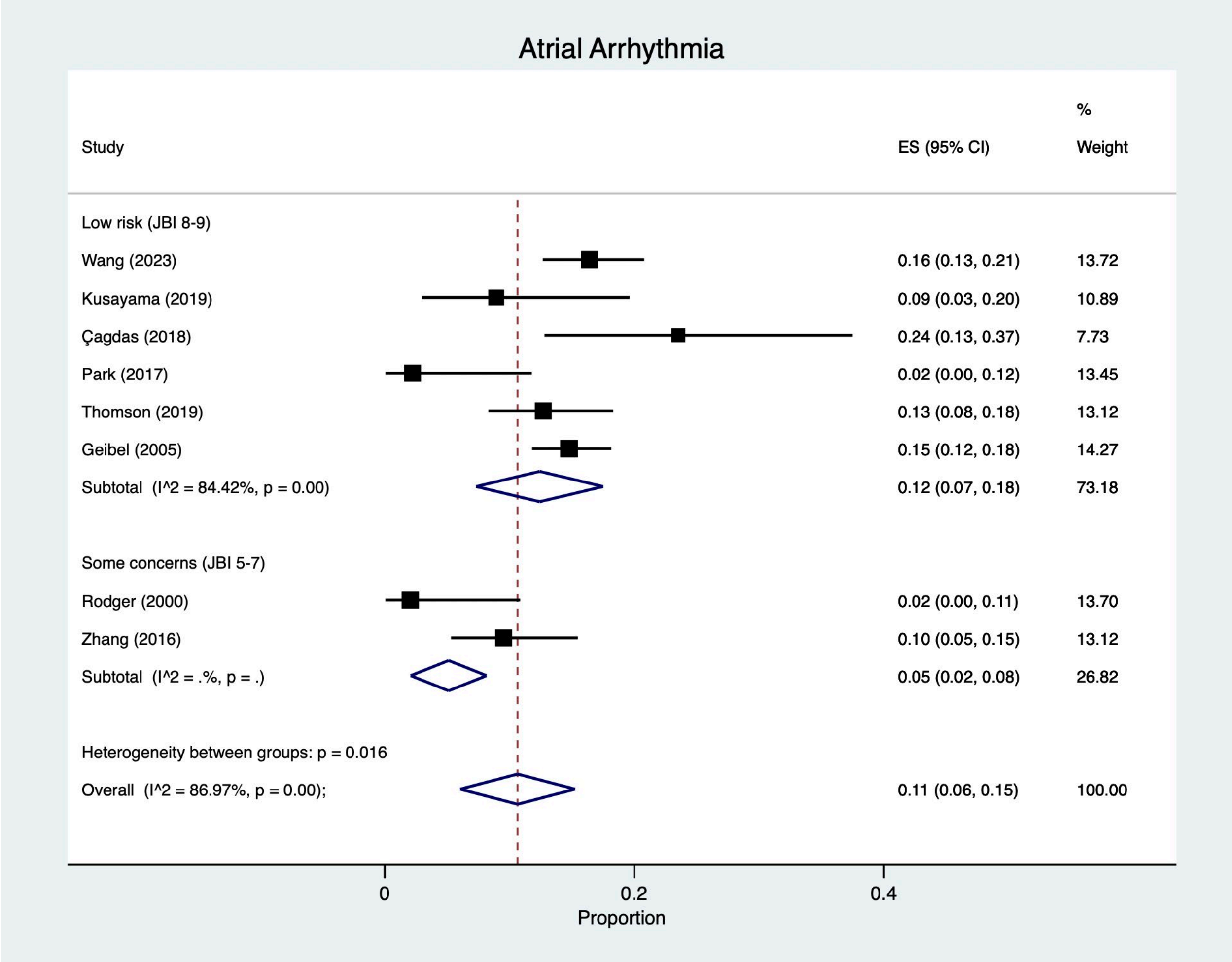

Figure S9. Forest plot of the prevalence of clockwise rotation in patients with acute pulmonary embolism

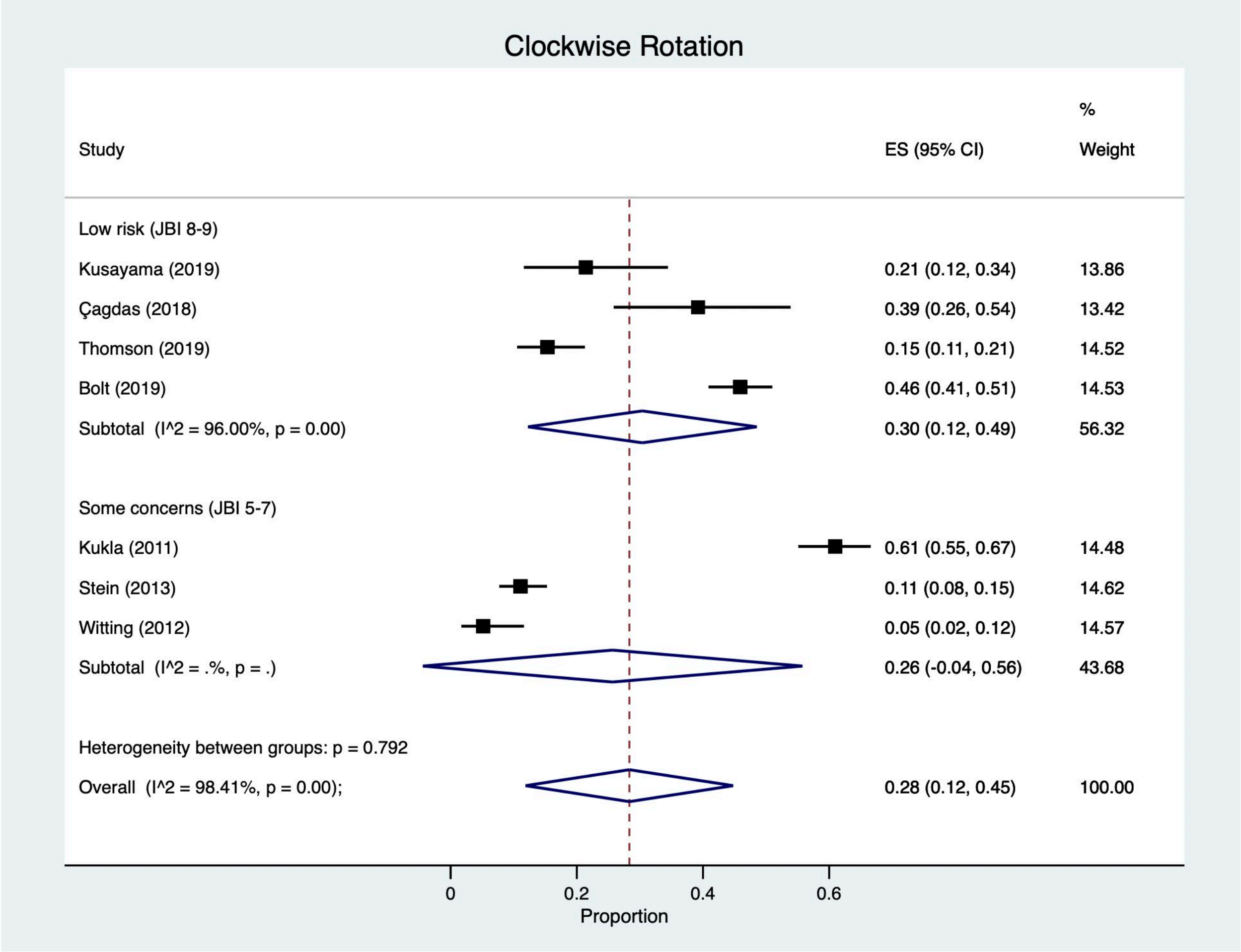

Figure S10. Forest plot of the prevalence of P pulmonale in patients with acute pulmonary embolism

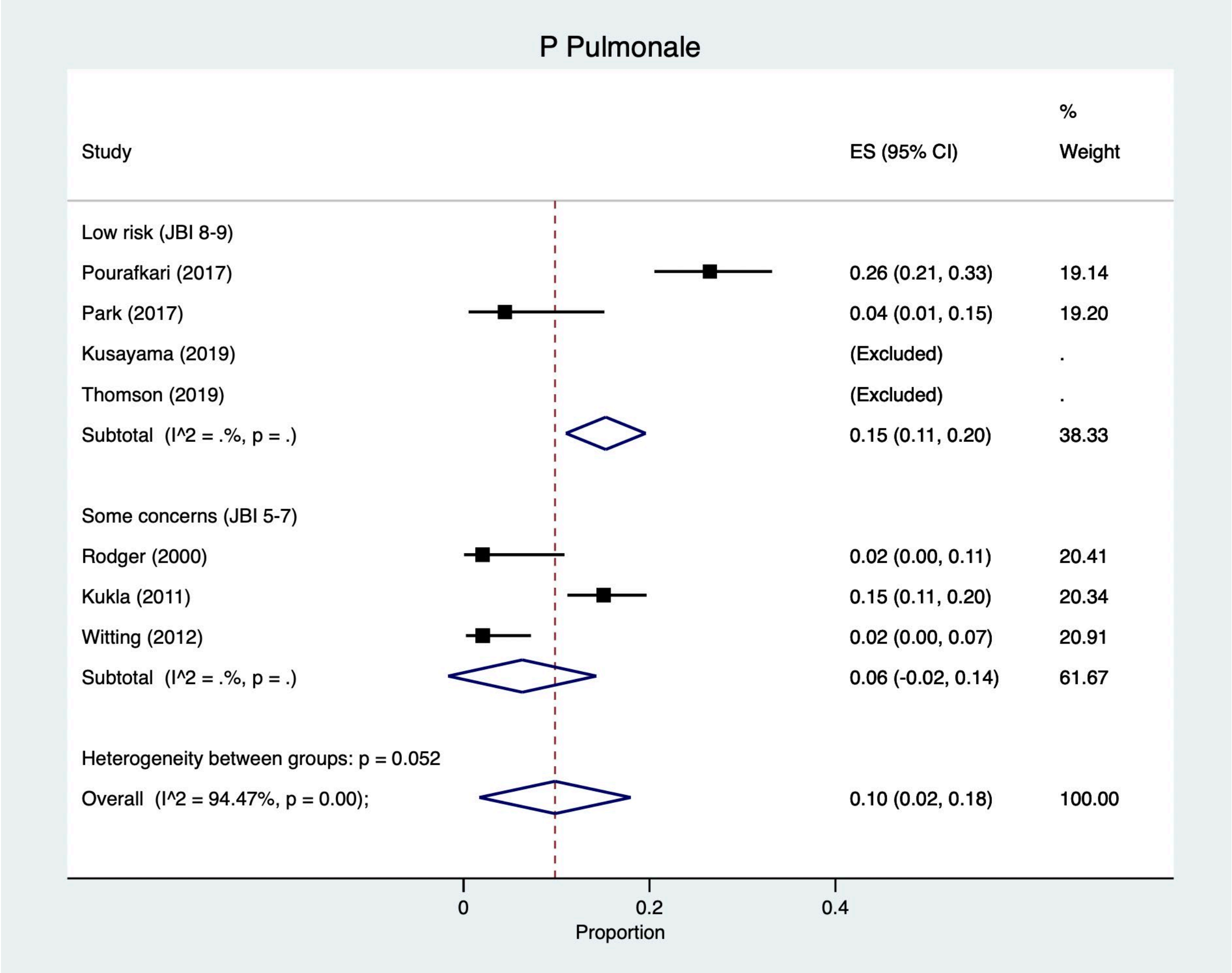

Figure S11. Forest plot of the prevalence of S1S2S3 in patients with acute pulmonary embolism

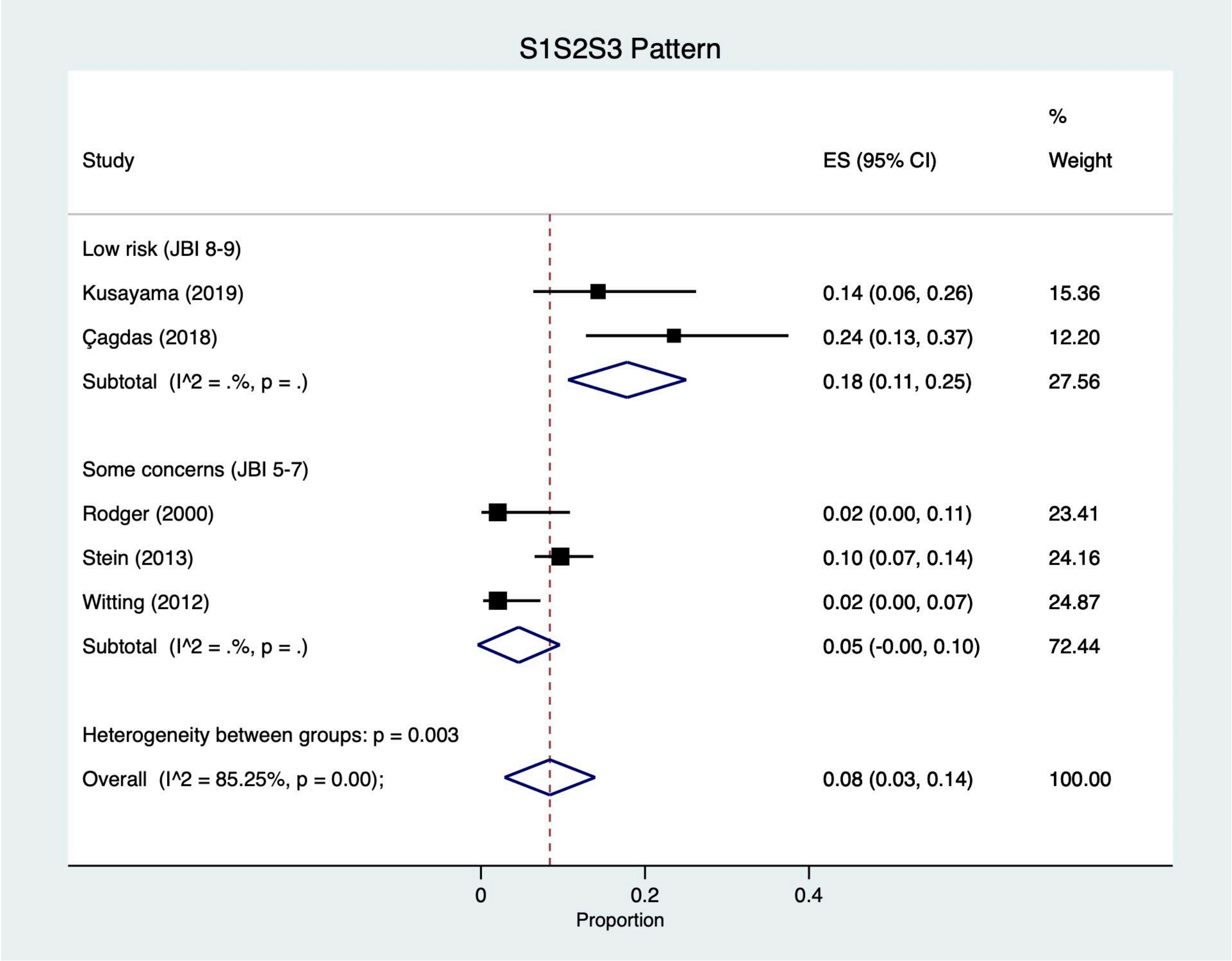

Figure S12. Funnel plot showing the publication bias among studies reporting the prevalence of S1Q3T3 in acute PE cases

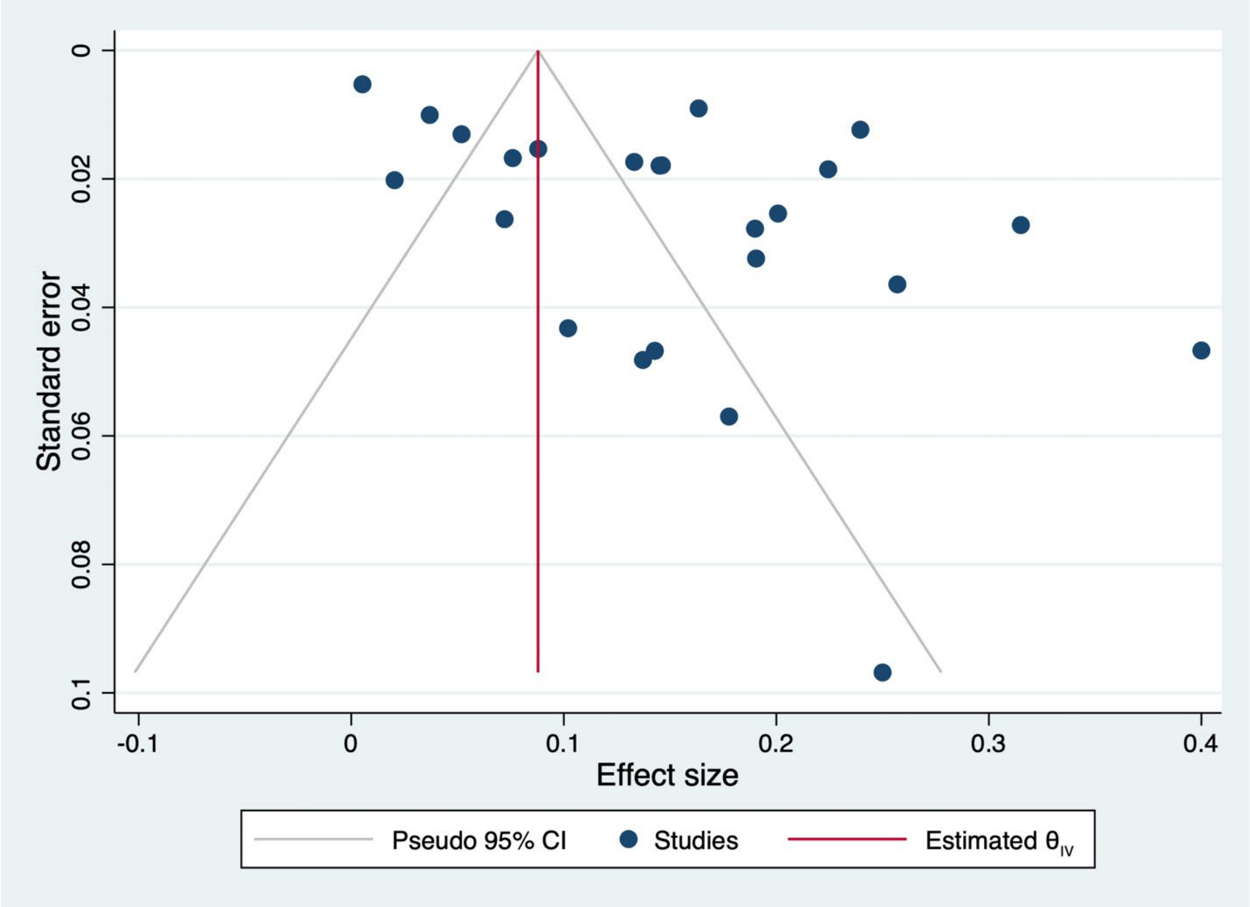

Figure S13. Funnel plot showing the publication bias among studies reporting the prevalence of right bundle branch block in acute PE cases

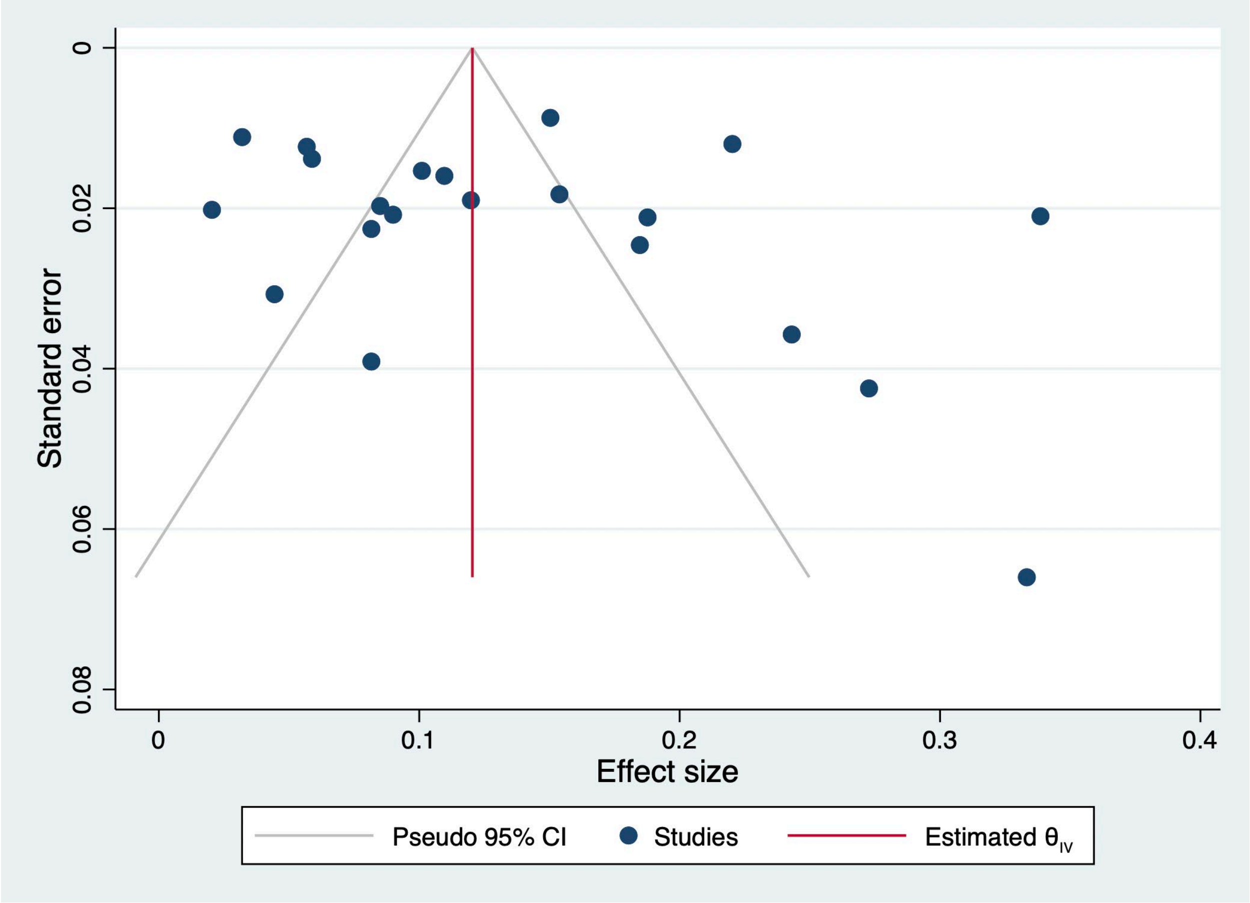

Supplement: Supplementary file 1 [file jcm-14-04750-s001.zip › jcm-3667676-supplementary.pdf]
